# Radiofrequency Irradiation Mitigated UV-B-Induced Skin Pigmentation by Increasing Lymphangiogenesis

**DOI:** 10.3390/molecules27020454

**Published:** 2022-01-11

**Authors:** Hyoung Moon Kim, Seyeon Oh, Kyung-A Byun, Jin Young Yang, Hye Jin Sun, Donghwan Kang, Kuk Hui Son, Kyunghee Byun

**Affiliations:** 1 Department of Anatomy & Cell Biology, College of Medicine, Gachon University, Incheon 21936, Korea; drmac12@me.com (H.M.K.); kabyun95@gmail.com (K.-A.B.); 2 Functional Cellular Networks Laboratory, Department of Medicine, Graduate School and Lee Gil Ya Cancer and Diabetes Institute, College of Medicine, Gachon University, Incheon 21999, Korea; seyeon8965@gmail.com (S.O.); roswellgirl111@gmail.com (J.Y.Y.); 3 Jeisys Medical Inc., Seoul 08501, Korea; sunhj@jeisys.com (H.J.S.); kang@jeisys.com (D.K.); 4 Department of Thoracic and Cardiovascular Surgery, Gil Medical Center, Gachon University, Incheon 21565, Korea

**Keywords:** dermal macrophage-containing melanin, lymphangiogenesis, radiofrequency microneedling, ultraviolet-B, skin pigmentation

## Abstract

Dermal macrophages containing melanin increase skin pigmentation since dermal melanin removal is slower than epidermal melanin removal. Lymphatic vessels are also involved in melanin clearance. We evaluated whether radiofrequency (RF) irradiation induced an increase in HSP90, which promotes lymphangiogenesis by activating the BRAF/MEK/ERK pathway and decreasing tyrosinase activity, in the UV-B exposed animal model. The HSP90/BRAF/MEK/ERK pathway was upregulated by RF. Tyrosinase activity and the VEGF-C/VEGFR 3/PI3K/pAKT1/2/pERK1/2 pathway, which increase lymphangiogenesis, as well as the expression of the lymphatic endothelial marker LYVE-1, were increased by RF. Additionally, the number of melanin-containing dermal macrophages, the melanin content in the lymph nodes, and melanin deposition in the skin were decreased by RF. In conclusion, RF increased HSP90/BRAF/MEK/ERK expression, which decreased tyrosinase activity and increased lymphangiogenesis to eventually promote the clearance of dermal melanin-containing macrophages, thereby decreasing skin pigmentation.

## 1. Introduction

Skin hyperpigmentation is usually accompanied by post-inflammatory hyperpigmentation or melasma [1]. In normal skin, melanin usually exists in the basal layer of the epidermis and is not exhibited in the dermis [2,3]. Skin inflammation induces either hyperplasia of epidermal melanocytes or increased function of epidermal melanocytes, which both result in enhanced melanogenesis and increased deposition of melanin in the epidermis or dermis [2,3,4,5,6]. Basement membrane damage induced by inflammation allows melanin to enter the dermis, where dermal macrophages phagocytose melanin through melanophagy [2,3]. Moreover, macrophages containing melanin travel to the epidermis, where macrophages further phagocytose melanosomes and then return to the dermis [2,3]. Through these processes, increased melanin deposition leads to hyperpigmentation [7]. It has not been fully revealed how melanin in the dermis is removed or what route is used to remove dermal melanin. However, the lymphatic system might be involved in the removal of melanin in the dermis. It was reported that hyperpigmented Kitl-Tg mice exhibited extreme proliferation of melanocyte in the epidermis, and their lymph nodes showed accumulation of melanin granules which were transported by Langerhans cells [7,8]. Interestingly, the accumulation rate of melanin granules was constant from 3 to 50 weeks [8]. Moreover, melanophages are frequently exhibited in the lymph nodes of patients with dermal pigmentation induced by inflammatory skin diseases [9,10]. Based on these results, melanin in the dermis might be transported through the lymphatic system.

The lymphatic system plays multiple essential functions in the maintenance of homeostasis, including the regulation of interstitial pressure, immune surveillance, and lipid metabolism [11]. Under normal circumstances, lymphatic endothelial cells (LECs) are generally quiescent. However, various inflammatory stimuli initiate LEC proliferation or lymphangiogenesis in inflamed tissues [12,13,14]. Lymphangiogenesis promotes the removal of cytokines, inflammatory cells, and antigens from inflamed tissue and facilitates recovery from inflammation [12,13,14]. Lymphangiogenesis induced by inflammation is mainly initiated by vascular endothelial growth factor (VEGF) A, VEGF-C, and VEGF-D through VEGF receptor 2 (VEGFR 2) and/or VEGFR 3 signaling [15,16,17,18,19]. VEGF-A mainly induces lymphangiogenesis through VEGFR 2; however, VEGF-C and VEGF-D promote lymphangiogenesis via VEGFR 2 and/or VEGFR 3 signaling [20]. Tyrosinase is an enzyme that is primarily involved in melanogenesis in melanocytes [21,22]. Tyrosinase is also involved in inhibiting lymphangiogenesis in the eyes [23]. The albino mutation is related to increased corneal hemangiogenesis [24]. Moreover, tyrosinase or tyrosinase products impede the migration of endothelial cells [24].

Radiofrequency (RF) involves the emission of electromagnetic radiation converted into thermal energy to generate controlled heat in deep skin tissues [25]. Increasing tissue temperature leads to vasodilation, which sequentially increases the resorption of intercellular fluids and promotes waste removal through the lymphatic system [26,27]. Epidermal and dermal cells consequently express heat shock proteins (HSPs) that sense various stresses and respond to stress to decrease stress-induced damage [28,29]. RF irradiation induces the expression of HSPs such as HSP47 or HSP72, which stimulate fibroblasts to increase collagen production [30,31]. HSP90 is an essential intracellular molecular chaperone that regulates numerous protein clients. HSP90 is involved in various cellular processes, including protein folding, development, immune response, and DNA repair [32]. HSP90 also upregulates protein kinase B (AKT) or extracellular signal-regulated kinase (ERK), which increases cell migration during wound healing or cancer metastasis [33]. The activation of AKT and ERK is essential for lymphangiogenesis mediated by VEGF-C or VEGF-D [34]. In fact, HSP90 production activates AKT, thus leading to lymphangiogenesis by promoting the migration and tube formation of LECs [35]. HSP90 is also involved in the upregulation of ERK through BRCA1-associated protein 2 (BRAP)/mitogen-activated protein/extracellular signal-regulated kinase (MEK) in melanomas [36]. Inhibitors of HSP90 and BRAF induce melanoma cell differentiation, which increases melanin synthesis [36]. The ERK pathway is also involved in regulating microphthalmia-associated transcription factor (MITF) in melanocytes [37,38]. ERK activation leads to the phosphorylation and degradation of MITF, which is the master regulator of tyrosinase expression [39,40]. Thus, activated ERK decreases melanogenesis by decreasing tyrosinase in melanocytes [41,42].

Although RF irradiation increases HSPs, whether RF irradiation decreases dermal pigmentation by modulating lymphangiogenesis has not yet been established. We evaluated whether RF irradiation increases HSP90 production, which sequentially leads to increased BRAF/MEK/ERK activity in the UV-B induced hyperpigmentation animal model in this study. It was hypothesized that ERK upregulation decreases tyrosinase activity and thereby attenuates the inhibition of lymphangiogenesis by tyrosinase. Enhanced lymphangiogenesis through tyrosinase inhibition leads to increased clearance of dermal melanophages and decreased skin pigmentation, especially in the dermis.

## 2. Results

### 2.1. RF Irradiation Increased the Expression of HSP90, BRAF, MEK, and ERK

**Figure 1:**
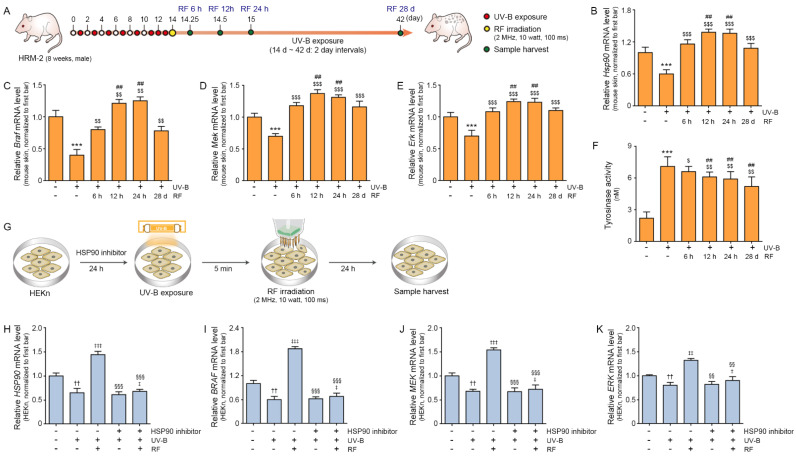
Effects of RF on the expression of *Hsp90*, *Braf*, *Mek*, and *Erk* in keratinocytes. (**A**) Diagram of the animal model used to validate the melanin reduction mechanism by lymphangiogenesis induced by RF irradiation. HRM-2 mice (eight weeks, male) were exposed to UV-B exposed for 5 min every other day for 10 days and 5 min per day from the 11th to 13th days. On the 14th day, RF irradiation was performed on UV-B exposed animal models for 100 ms at 10 W and 2 MHz. Mice were euthanized, and skin tissue specimens were obtained 6 h (14.25th day), 12 h (14.5th day), 24 h (15th day), and 28 d (42nd day) after RF irradiation. (**B**–**E**) The mRNA expression levels of *Hsp90*, *Braf*, *Mek*, and *Erk* were determined in skin tissue. The expression of *Hsp90* (**B**), *Braf* (**C**), *Mek* (**D**), and *Erk* (**E**) decreased in the UV-B group and increased in the RF irradiated group. All mRNA levels were measured using qRT–PCR, normalized versus *Actb*, and expressed relative to the levels in the control group (first bar). (**F**) Tyrosinase activity was evaluated using ELISA in skin tissues. Tyrosinase activity increased in the UV-B group and decreased in the RF irradiated group. (**G**) Schematic diagram confirming the regulatory effect of RF on the expression of HSP90 in HEKn. HSP90 inhibitor (17-AAG) was used to treat HEKn. After 24 h, HEKn were exposed to UV-B for 5 min. Then, RF was irradiated for 100 ms at 10 W and 2 MHz, and samples were harvested 24 h later. (**H**–**K**) The mRNA expression levels of *HSP90* (**H**)*, BRAF* (**I**)*, MEK* (**J**) and *ERK* (**K**) decreased in the UV-B group and increased in the RF irradiated group. However, the increasing effect of RF disappeared with HSP90 inhibitor treatment. All mRNA levels were measured using qRT–PCR, normalized versus ACTB, and expressed relative to the levels in the control group (first bar). Data are presented as the mean ± SD. ***, *p* < 0.001 vs. UV-B(-)/RF(-); $, *p* < 0.05, $$, *p* < 0.01, and $$$, *p* < 0.001 vs. UV-B(+)/RF(-); ##, *p* < 0.01 vs. UV-B(+)/RF 6 h; ††, *p* < 0.01 vs. HSP90 inhibitor(-)/UV-B(-)/RF(-); ‡, *p* < 0.05, ‡‡, *p* < 0.01, and ‡‡‡, *p* < 0.001, vs. HSP90 inhibitor(-)/UV-B(+)/RF(-); §§, *p* < 0.01, and §§§, *p* < 0.001 vs. HSP90 inhibitor(-)/UV-B(+)/RF(+). BRAF, serine/threonine-protein kinase B-Raf; d, day; ERK, extracellular signal-regulated kinase; ELISA, enzyme-linked immunosorbent assay; h, hours; HEKn, human epidermal keratinocyte; HSP90, heat shock protein 90; MEK, mitogen-activated protein/extracellular signal-regulated kinase; MHz, megahertz; ms, milliseconds; RF, radiofrequency; UV-B, ultraviolet-B.

First, we evaluated whether RF affects the expression of *Hsp90*, *Braf*, *Mek*, and *Erk* in UV-B exposed animal skin. The mouse skin hyperpigmentation model was established by exposure to UV-B for 5 min every two days (d) for 10 d and then for 5 min every day for the following 3 d [43]. On day 14 after UV-B exposure was started, mice in the UV-B/RF group were irradiated to RF (2 MHz, 10 W, 100 ms) once. After RF irradiation, UV-B exposure was applied every 2 d for 28 d. In the UV-B/RF group, skin harvesting was performed at 6 hours (h), 12 h, 24 h, and 28 d after RF irradiation. In the control and UV-B groups, skin harvesting was performed at the end of the experiment, which coincided with the 28th day after RF irradiation in the UV-B/RF group (Figure 1A).

The expression levels of *Hsp90*, *Braf*, *Mek*, and *Erk* in the skin of the UV-B group were significantly lower than those in the control group at the end of the experiment. However, the expression levels of *Hsp90*, *Braf*, *Mek* and *Erk* in the UV-B/RF group at 6 h, 12 h, 24 h, and 28 d after RF irradiation were higher than those of the UV-B group. The expression levels of *Hsp90*, *Braf*, *Mek*, and *Erk* in the UV-B/RF group at 6 h after RF irradiation were significantly lower than those at 12 h and 24 h; however, the expression at 6 h was not significantly different from that at 28 d after RF irradiation (Figure 1B–E).

Next, we evaluated whether increased expression of BRAF/MEK/ERK lead to decrease tyrosinase activity and eventually induced the inhibition of lymphangiogenesis by tyrosinase.

The tyrosinase activity in the skin of the UV-B group was significantly higher than that in the control group at the end of the experiment. The tyrosinase activity in the skin of the UV-B group was significantly higher than that of the UV-B/RF group at 6 h, 12 h, 24 h, and 28 d after RF irradiation. The tyrosinase activity of the UV-B/RF group at 6 h was significantly higher than that of the UV-B/RF group at 12 h, 24 h, and 28 d (Figure 1F).

Since the expression of *Hsp90*, *Braf*, *Mek*, and *Erk* was increased by RF in the animal model, we tried to confirm whether RF irradiation could increase the expression of *HSP90* and whether HSP90 was a true upstream signaling pathway of BRAF, MEK, and ERK with an in vitro model. First, we evaluated whether RF modulates the expression of *HSP90* in UV-B exposed human primary epidermal keratinocytes (HEKn). Next, we also evaluated whether the modulatory effect of RF on the expression of HSP90 was changed after treatment with an HSP90 inhibitor (17-AAG) (Figure 1G).

The expression levels of *HSP90, BRAF, MEK,* and *ERK* were significantly decreased by UV-B. Those expression levels were increased by RF irradiation. The expression levels of *HSP90*, *BRAF*, *MEK*, and *ERK* in UV-B/HSP90 inhibitor treated HEKn were not significantly different from those in UV-B radiated HEKn. The expression levels of *HSP90, BRAF*, *MEK*, and *ERK* in UV-B/HSP90 inhibitor treated HEKn were also increased by RF irradiation. However, the increasing amount by RF was more prominent in the UV-B/RF treated HEKn than UV-B/HSP90 inhibitor/RF treated HEKn (Figure 1H–K).

Thus, these results suggested that RF involved increased expression of HSP90 and that HSP90 leads to the upregulation of BRAF, MEK, and ERK. When the HSP90 inhibitor was administered, the increasing effect of RF was decreased.

### 2.2. RF Upregulation of the VEGF-C/VEGFR 3/PI3K/pAKT/pERK Lymphangiogenesis Signaling Pathway in UV-B-Irradiated Skin

RF irradiation leads to the increased expression of ERK and decreased tyrosinase activity in UV-B exposed animal skin. Thus, we evaluated whether these changes by RF lead to increased expression of VEGF-C and VEGFR 3 in UV-B exposed animal skin.

**Figure 2:**
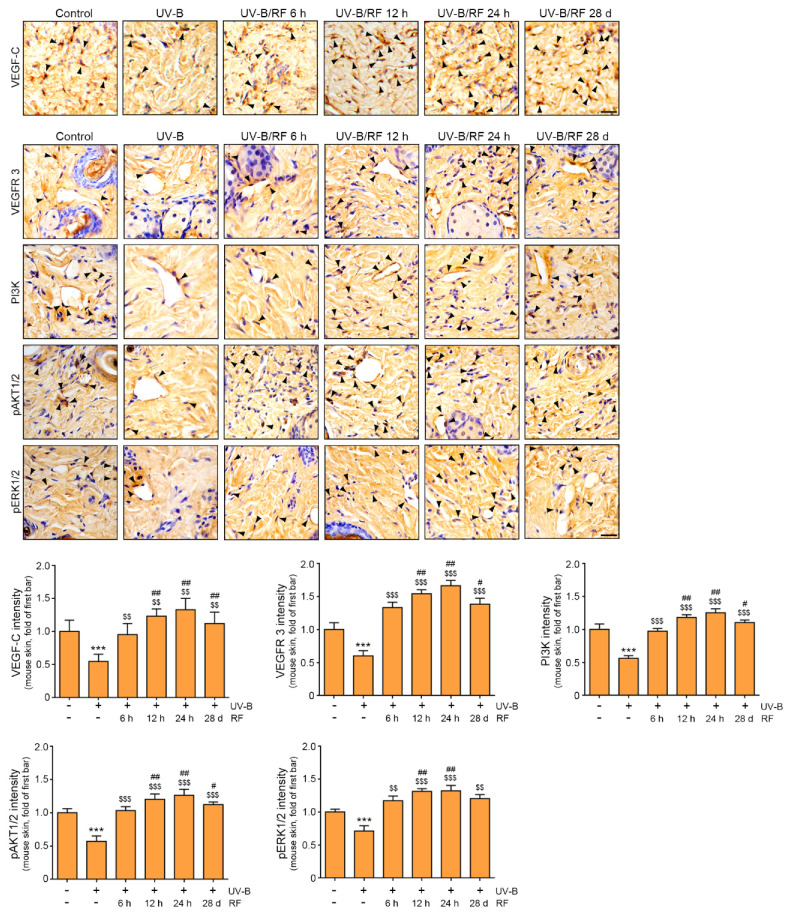
Upregulation of the lymphangiogenesis signaling pathway by RF irradiation of skin in the UV-B exposed animal model. Mouse skins were irradiated either with UV-B (UV-B) or with UV-B plus RF (UV-B/RF) or were otherwise unirradiated (control). (**A**) VEGF-C expression (arrows) in the dermis of UV-B exposed mouse skin as confirmed by immunohistochemistry (scale bar = 100 μm). (**B**) VEGFR 3, PI3K, pAKT1/2 and pERK1/2 expression (arrows) in the dermis of UV-B exposed mouse skin as confirmed by immunohistochemistry (scale bar = 100 μm). (**C**) Quantitative graph of representative VEGF-C images. The intensity of VEGF-C decreased in the UV-B group and increased in the RF irradiated group. (**D**–**G**) Quantitative graph of representative VEGFR 3, PI3K, pAKT1/2 and pERK1/2 images. The intensities of VEGFR 3, PI3K, pAKT1/2 and pERK1/2 decreased in the UV-B group and increased in the RF irradiated group. All intensities were analyzed against the level of the control group. Data are presented as the mean ± SD. ***, *p* < 0.001 vs. UV-B(-)/RF(-); $$, *p* < 0.01, and $$$, *p* < 0.001 vs. UV-B(+)/RF(-); #, *p* < 0.05, and ##, *p* < 0.01 vs. UV-B(+)/RF 6 h. d, day; h, hours; pAKT1/2, phosphorylated protein kinase B 1/2; pERK1/2, phosphorylated extracellular signal-regulated kinase 1/2; PI3K, phosphatidylinositol 3-kinase; RF, radiofrequency; UV-B, ultraviolet-B; VEGF-C, vascular endothelial growth factor C; VEGFR 3, VEGF receptor 3.

VEGF-C expression in the UV-B group was significantly lower than that in the control group at the end of the experiment. VEGF-C expression in the UV-B group was significantly lower than that in the UV-B/RF group at 6 h, 12 h, 24 h, and 28 d after RF irradiation. VEGF-C expression in the UV-B/RF group at 12 h, 24 h, and 28 d after RF irradiation was significantly higher than that at 6 h after RF irradiation (Figure 2A,C).

VEGFR 3 expression in the UV-B group was significantly lower than that in the control group at the end of the experiment. VEGFR 3 expression in the UV-B group was significantly lower than that in the UV-B/RF group at 6 h, 12 h, 24 h and 28 d after RF irradiation. VEGFR 3 expression in the UV-B/RF group at 12 h, 24 h and 28 d after RF was significantly higher than that at 6 h after RF irradiation (Figure 2B,D).

After binding of VEGF-C to VEGFR 3, the downstream signals of PI3K/AKT and ERK are activated, which leads to lymphangiogenesis [34]. Thus, we evaluated the changes in the expression of PI3K, pAKT1/2, and pERK1/2 by RF. The expression levels of PI3K, pAKT1/2, and pERK1/2 in the UV-B group were significantly lower than those in the control group at the end of the experiment. The expression levels of PI3K, pAKT1/2, and pERK1/2 in the UV-B group were significantly lower than those in the UV-B/RF group at 6 h, 12 h, 24 h, and 28 d after RF irradiation. The expression levels of PI3K and pAKT1/2 in the UV-B/RF group at 6 h were significantly lower than those in the UV-B/RF group at 12 h, 24 h, and 28 d after RF irradiation. The expression of pERK1/2 in the UV-B/RF group at 6 h was significantly lower than that in the UV-B/RF group at 12 h and 24 h after RF (Figure 2B,E–G).

These results suggested that RF involved upregulation of VEGF-C, VEGFR 3, PI3K, pAKT1/2 and pERK1/2 which are known as main signal pathway of lymphangiogenesis.

### 2.3. RF Irradiation Increased Lymphangiogenesis and Regulated the Permeability of Lymphatic Vessels

**Figure 3:**
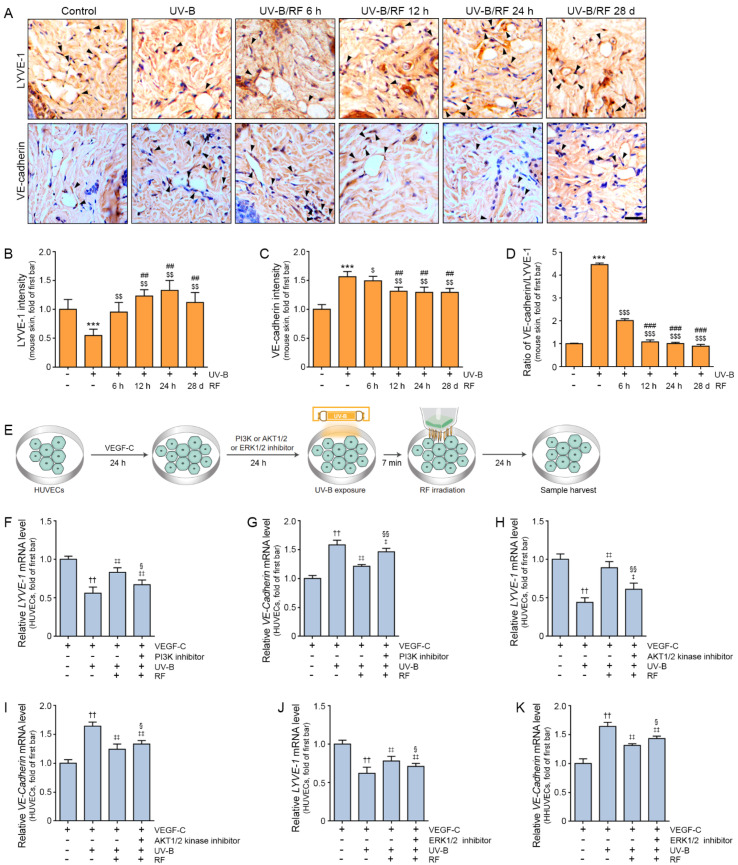
Modulatory effect of RF irradiation on lymphangiogenesis and the permeability of lymphatic vessels in the skin of the UV-B exposed animal model. Mouse skins were irradiated either with UV-B (UV-B) or with UV-B plus RF (UV-B/RF) or were otherwise unirradiated (control). (**A**) LYVE-1 (lymphangiogenesis marker) and VE-cadherin expression (permeability of lymphatic vessels) in the dermis of UV-B exposed mouse skin as confirmed by immunohistochemistry (scale bar = 100 μm). (**B**–**D**) Quantitative graphs of representative LYVE-1 and VE-cadherin images. The intensity of LYVE-1 decreased in the UV-B group and increased in the RF irradiated group (**B**). The intensity of VE-cadherin (**C**) and the ratio of LYVE-1/VE-cadherin (**D**) increased in the UV-B group and decreased in the RF irradiated group. (**E**) Schematic diagram confirming whether RF irradiation leads to lymphangiogenesis through the PI3K/AKT1/2 or ERK1/2 pathway in HUVECs. HUVECs were treated with VEGF-C for 24 h, and then inhibitors of PI3K (LY294002), AKT1/2 (AKT1/2 kinase inhibitor), and ERK1/2 (U0126) were administered for 24 h. Next, HUVECs were exposed to UV-B for 7 min, RF irradiation was administered for 100 ms at 10 W and 2 MHz, and the samples were harvested 24 h later. (**F**–**K**) The mRNA expression levels of *LYVE-1* (**F**,**H**,**J**) decreased in the UV-B group and increased in the RF irradiated group. However, the increasing effect of RF disappeared with PI3K, AKT1/2, and ERK1/2 inhibitor treatment. The expression level of *VE-cadherin* (**G**,**I**,**K**) increased in the UV-B group and decreased in the RF-irradiated group. However, the decreasing effect of RF disappeared with PI3K, AKT1/2, and ERK1/2 inhibitor treatment. All mRNA levels were measured using qRT–PCR, normalized versus ACTB, and expressed relative to the levels in the control group (first bar). Data are presented as the mean ± SD. ***, *p* < 0.001 vs. UV-B(-)/RF(-); $, *p* < 0.05, $$, *p* < 0.01, and $$$, *p* < 0.001 vs. UV-B(+)/RF(-); ##, *p* < 0.01, and ###, *p* < 0.001 vs. UV-B(+)/RF 6 h; ††, *p* < 0.01 vs. VEGF-C(+)/inhibitor(-)/UV-B(-)/RF(-); ‡, *p* < 0.05, and ‡‡, *p* < 0.01, vs. VEGF-C(+)/inhibitor(-)/UV-B(+)/RF(-); §, *p* < 0.05, and §§, *p* < 0.01 vs. VEGF-C(+)/inhibitor(-)/UV-B(+)/RF(+). AKT1/2 inhibitor, protein kinase B 1/2 inhibitor; d, day; ERK1/2 inhibitor, extracellular signal-regulated kinase 1/2 inhibitor; h, hours; LYVE-1, lymphatic vessel endothelial hyaluronan receptor 1; PI3K inhibitor, phosphatidylinositol 3-kinase inhibitor; RF, radiofrequency; UV-B, ultraviolet-B; VE-cadherin, vascular endothelial-cadherin; VEGF-C, vascular endothelial growth factor C.

Lymphatic vessel endothelial hyaluronan receptor 1 (LYVE-1) expression (a marker of LECs) in the UV-B group was significantly lower than that in the control group at the end of the experiment. The expression of LYVE-1 in the UV-B group was significantly lower than that in the UV-B/RF group at 6 h, 12 h, 24 h, and 28 d after RF irradiation. The expression of LYVE-1 at 12 h, 24 h, and 28 d was significantly higher than that at 6 h after RF irradiation (Figure 3A,B).

The permeability of lymphatic vessels was evaluated by determining VE-cadherin expression. Even though VE-cadherin is a vital molecule for vascular structure formation [44], its overexpression decreases lymphatic permeability, which leads to decreased lymphatic drainage [45]. VE-cadherin expression in the UV-B group was significantly higher than that in the control group at the end of the experiments (Figure 3A,C). The expression of VE-cadherin in the UV-B group was significantly higher than that in the UV-B/RF group at 6 h, 12 h, 24 h, and 28 d. The expression of VE-cadherin at 12 h, 24 h, and 28 d was significantly lower than that at 6 h (Figure 3A,C).

The ratio of VE-cadherin to LYVE-1 in the UV-B group was significantly higher than that in the control group at the end of the experiments (Figure 3D). The ratio of VE-cadherin to LYVE-1 in the UV-B group was significantly higher than that in the UV-B/RF group at 6 h, 12 h, 24 h, and 28 days. The ratio of VE-cadherin to LYVE-1 in the UV-B/RF group at 12 h, 24 h, and 28 days was significantly lower than that at 6 h (Figure 3D).

These results showed that RF irradiation leads to upregulation of LYVE-1, which suggests increased lymphangiogenesis. RF also decreased VE-cadherin, which suggested that RF is involved in regulating the permeability of lymphatic vessels.

Next, we evaluated whether RF irradiation leads to lymphangiogenesis through the PI3K/AKT1/2 or ERK1/2 pathway by treating UV-B exposed HUVECs with VEGF-C and inhibitors of PI3K (LY294002), AKT1/2 (AKT1/2 kinase inhibitor), and ERK1/2 (U0126) (Figure 3E).

The expression of LYVE-1 in HUVECs was significantly decreased by VEGF-C treatment and UV-B exposure; however, it was increased by RF irradiation. The increasing effect of RF on the expression of LYVE-1 was decreased by treatment with a PI3K inhibitor. The expression of VE-cadherin was increased by VEGF-C and UV-B in HUVECs. However, it was decreased by RF. The decreasing effect of RF on the expression of VE-cadherin was decreased when the PI3K inhibitor was added (Figure 3F,G).

The expression of LYVE-1 was significantly decreased by VEGF-C and UV-B and increased by RF. The increasing effect of RF on the expression of LYVE-1 was decreased by treatment with AKT1/2 and ERK1/2 inhibitors. The expression of VE-cadherin was increased by VEGF-C and UV-B in HUVECs. However, it was decreased by RF irradiation. The decreasing effect of RF on the expression of VE-cadherin was decreased when AKT1/2 or ERK1/2 inhibitor treatment was administered (Figure 3H–K).

These results suggested that the effect of RF irradiation on increasing the expression of LYVE-1 and decreasing the expression of VE-cadherin was induced by the increased expression of PI3K/AKT and ERK.

### 2.4. RF Irradiation of the Skin Decreased the Levels of Melanin-Containing Macrophages

**Figure 4:**
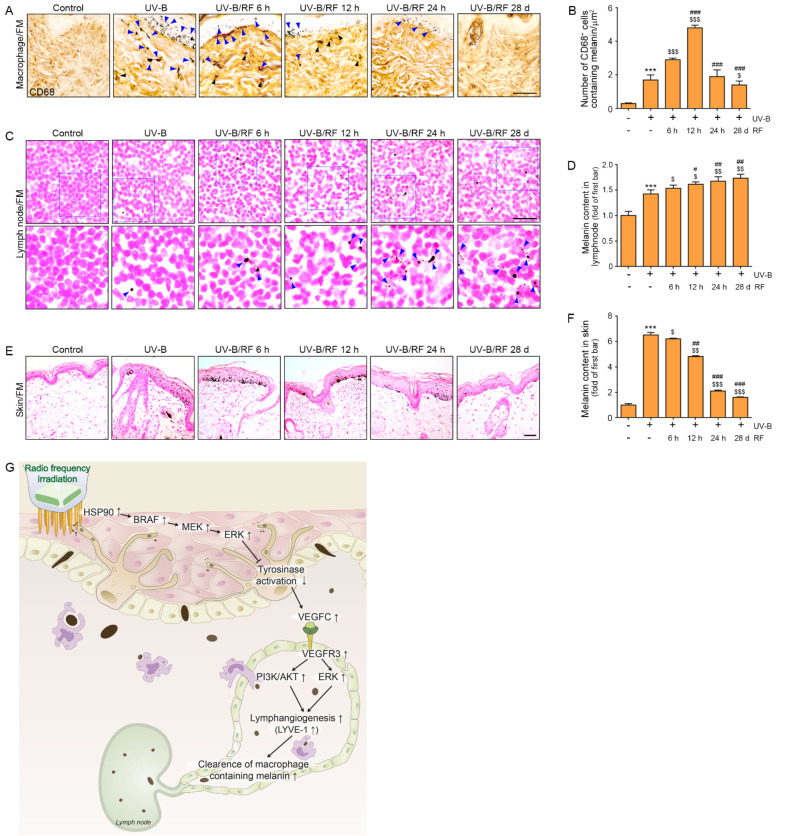
Attenuation of the number of macrophages containing melanin by RF irradiation in the dermis in the UV-B exposed animal model. Mouse skins were irradiated either with UV-B (UV-B) or with UV-B plus RF (UV-B/RF) or were otherwise unirradiated (control). (**A**) CD68/melanin (blue arrows, melanin; black arrows, macrophages containing melanin) expression in the dermis of UV-B exposed mouse skin as confirmed by immunohistochemistry and Fontana–Masson (FM) staining. (scale bar = 100 μm (**B**) Quantitative graphs of representative CD68/FM images. The number of CD68^+^ cells containing melanin increased in the UV-B group and more increased in the RF irradiated group than UV-B group. (**C**) The melanin content in the lymph nodes (blue arrows) as determined by FM staining (scale bar = 100 μm). (**D**) Quantitative graphs of representative FM in lymph node images. The melanin content in the lymph nodes increased in the UV-B group and increased in the RF irradiated group. (**E**) The melanin content in skin as determined by FM staining (scale bar = 100 μm). (**F**) Quantitative graphs of representative FM in skin images. The melanin content in the skin increased in the UV-B group and decreased in the RF irradiated group. (**G**) Summary of this study. Data are presented as the mean ± SD. ***, *p* < 0.001 vs. UV-B(-)/RF(-); $, *p* < 0.05, $$, *p* < 0.01, and $$$, *p* < 0.001 vs. UV-B(+)/RF(-); #, *p* < 0.05, ##, *p* < 0.01, and ###, *p* < 0.001 vs. UV-B(+)/RF 6 h. CD68, cluster of differentiation 68; d, day; FM, Fontana–Masson; h, hours; RF, radiofrequency; UV-B, ultraviolet-B.

To evaluate macrophages containing melanin, co-staining with Fontana–Masson (FM) staining (a melanin marker) and CD68 (a macrophage marker) was conducted. The number of melanin-containing macrophages in the UV-B group was significantly higher than that in the control group or UV-B/RF group at the end of the experiments. The number of melanin-containing macrophages in the UV-B group was significantly lower than that in the UV-B/RF group at 6 h and 12 h after RF irradiation. The number of melanin-containing macrophages in the UV-B/RF group at 6 h was significantly lower than in the UV-B/RF group at 12 h, however it was significantly higher than those of UV-B/RF at 24 h and 28 d (Figure 4A,B).

Thus, those results showed that RF decreased melanin-containing macrophages in the skin.

### 2.5. RF Irradiation Increased the Melanin Content in Lymph Nodes and Decreased Skin Pigmentation

The melanin content in the lymph nodes was evaluated to determine whether melanin could be cleared through the lymphatic vasculature. The melanin content in the lymph nodes of the UV-B group was significantly higher than that of the control at the end of the experiment. The melanin content in the lymph nodes of the UV-B group was lower than that of the UV-B/RF group at 6 h, 12 h, 24 h, and 28 d. The melanin content in the lymph nodes of the UV-B/RF group at 6 h was significantly lower than those of the UV-B/RF group at 12 h, 24 h, and 28 d (Figure 4C,D).

Melanin deposition in the skin in the UV-B group was significantly higher than that in the control group at the end of the experiments. The melanin deposition in the skin of the UV-B group was higher than those of the UV-B/RF group at 6 h, 12 h, 24 h, and 28 d. The melanin deposition in the skin of the UV-B group at 6 h was significantly higher than those of the UV-B/RF group at 12 h, 24 h, and 28 d (Figure 4E,F).

Thus, those results showed that RF increased melanin clearance through lymphatic vessels and decreased skin pigmentation.

## 3. Discussion

Excessive melanin production by inflammation in the epidermis is easier to degrade and faster to eliminate than dermal melanin. Moreover, melanin phagocytosed in the dermis by macrophages is degraded more slowly than that in the epidermis and induces skin pigmentation [46].

Since the eyes lack lymphatic vessels, tyrosinase expression would thus possibly be associated with ocular alymphacity and immune privilege, as well as melanin production [47,48]. Tyrosinase deficient C57BL/6N mice (albino B6N-Tyrc-Brd) showed significantly higher limbal and corneal lymphatic surface areas, as indicated by LYVE-1 expression, than tyrosinase intact C57BL/6N mice [23]. Additionally, tyrosinase-deficient mice also showed more significant inflammation-induced lymphangiogenesis than tyrosinase-intact mice [23]. HSP90 upregulates ERK, which in turn downregulates tyrosinase [37,38]. Moreover, HSP90 induces lymphangiogenesis through AKT, which is a downstream target of VEGFR 3 [35].

In this study, we evaluated whether RF irradiation increased HSP90 expression, which would activate the BRAF/MEK/ERK pathway and consequently decrease tyrosinase activity. HSP90/BRAF/MEK/ERK expression was significantly decreased by UV-B and increased by RF irradiation in UV-B exposed mouse skin. The increasing effect of RF was highest at 12 h and 24 h after RF irradiation. An in vitro model in which HEKn were exposed to UV-B also showed that HSP90/BRAF/ERK expression was decreased by UV-B and increased by RF irradiation. However, the increase effect by RF on HSP90/BRAF/MEK/ERK expression was decreased by HSP90 inhibitor treatment. Thus, RF increased HSP90, and HSP90 is involved in the upregulation of the BRAF/MEK/ERK pathway.

Tyrosinase activity, which was increased by UV-B exposure, was, conversely, decreased by RF irradiation of UV-B exposed mouse skin in our study. Additionally, the expression of VEGF-C and VEGFR 3 was significantly decreased by UV-B but increased by RF irradiation in the skin. VEGFR 3 binds with VEGF-C, thereby activating AKT or ERK to induce the proliferation and migration of LECs and promote lymphatic vessel formation [49]. In our study, the expression of PI3K/pAKT1/2/pERK1/2 was decreased by UV-B in mouse skin, and PI3K/pAKT1/2/pERK1/2 expression was increased by RF.

We also evaluated lymphatic vessel formation by staining for LYVE-1, which is a marker of LECs. LYVE-1 expression was decreased by UV-B but increased by RF in UV-B exposed skin. UV-B exposure also led to decreased LYVE-1 expression in HUVECs. The expression of LYVE-1 was increased by RF; however, increased expression by RF was not shown when HUVECs were treated with inhibitors of PI3K, AKT1/2, or ERK1/2. Thus, RF led to increased expression of LYVE-1 through PI3K, AKT, and ERK.

Maintaining lymphatic permeability is also essential to lymphatic drainage [45]. Here, we evaluated lymphatic permeability with the expression of VE-cadherin. VE-cadherin expression was significantly increased by UV-B and decreased by RF irradiation in both mouse skin and HUVECs. The decreasing effect of RF disappeared after treatment with inhibitors of PI3K, AKT1/2, or ERK1/2. Since inhibitors of PI3K, AKT1/2, or ERK1/2 have non-specific off-target effects, we could not conclude that PI3K, AKT1/2, or ERK1/2 are only pathway which involve in increasing lymphangiogenesis by RF. However, our results showed that RF involved in decreasing VE-cadherin through PI3K, AKT, and ERK.

Thus, RF irradiation seems to promote lymphangiogenesis and maintain lymphatic permeability. The lymphatic system is involved in the removal of dermal melanin. We evaluated whether melanin containing macrophages in the dermis were decreased by RF. In the dermal area, melanin-containing cells, which were positive for CD68, were increased by UV-B exposure. Those melanin-containing CD68 cells were increased at 6 h after RF irradiation more than those of UV-B group, however it decreased from 24 h after RF irradiation.

We thought that dermal melanin-containing CD68 cells would be eliminated through the lymphatic system. Thus, we evaluated whether the melanin content in the lymph nodes was changed by RF. The melanin content in the lymph nodes was increased by RF. In addition, melanin deposition in the skin was also increased by UV-B and decreased with RF irradiation in mouse skin.

Our study showed that increased HSP90 induced by RF irradiation upregulated BRAF/MEK/ERK and eventually decreased tyrosinase activity. By decreasing the inhibitory effect of tyrosinase on lymphangiogenesis, lymphatic vessel formation and sustained permeability of lymphatics led to the increasing clearance of dermal macrophages containing melanin. These changes eventually lead to decreased melanin deposition in the skin (Figure 4G).

## 4. Materials and Methods

### 4.1. In Vitro Model and RF Irradiation

HEKn (Cat. PCS-200-011; ATCC, Manassas, VA, USA) were cultivated with dermal cell basal medium (Cat. PCS-200-030; ATCC) and a keratinocyte growth kit (Cat. #PCS-200-040; ATCC). HEKn were maintained at 37 °C under 5% CO_2_ in a state of proliferation and nondifferentiation. For the in vitro model, the cells were exposed to UV-B for 5 min, irradiated with RF (POTENZA, Jeisys Medical Inc., Seoul, Korea; 2 MHz, 10 W, 100 ms), and cultured for 24 h.

HUVECs (Cat. CEFO-HUVEC; CEFO Bio, Seoul, Korea) were cultivated with a HUVEC growth medium kit (Cat. CEFOgro-HUVEC; CEFO Bio). The HUVECs were maintained at 37 °C under 5% CO_2_. For the in vitro model, the cells were exposed to UV-B for 7 min, irradiated with RF (POTENZA, Jeisys Medical Inc.; 2 MHz, 10 W, 100 ms), and cultured for 24 h.

### 4.2. Inhibition Study

#### 4.2.1. HSP90 Inhibition Study on HEKn

HEKn were treated with an HSP90 inhibitor (17-AAG; Cat. 11039, Cayman, Ann Arbor, MI, USA) at 0.5 µM for 24 h at 37 °C under 5% CO_2_. After 24 h, the cells were exposed to UV-B for 5 min, irradiated with RF (POTENZA, Jeisys Medical Inc.; 2 MHz, 10 W, 100 ms), and cultured for 24 h at 37 °C under 5% CO_2_ (Figure 1G).

#### 4.2.2. PI3K, AKT, and ERK Inhibition Study on HUVECs

HUVECs were treated with VEGF-C (Cat. 9199-VC, R&D systems, Minneapolis, MN, USA) at 100 ng/mL. After 24 h, the cells were treated with a PI3K inhibitor (LY294002; Cat. #440202, Sigma Aldrich, St. Louis, MO, USA) at 1 µM, AKT1/2 inhibitor (AKT1/2 kinase inhibitor; Cat. #A6730, Sigma Aldrich) at 40 µM, and ERK1/2 inhibitor (U0126; Cat. #U0120, Sigma Aldrich) at 50 µM for 24 h. Then, HUVECs treated with VEGF-C and inhibitor were exposed to UV-B for 7 min, irradiated with RF (POTENZA, Jeisys Medical Inc.; 2 MHz, 10 W, 100 ms), and cultured for 24 h at 37 °C under 5% CO_2_ (Figure 3E).

### 4.3. In Vivo Model and RF Irradiation

This study was approved by Gachon University and executed in accordance with the guidelines of the Institutional Animal Care and Use Committee (Approval Number LCDI-2020-0115). The animal experiments were conducted at the Center of Animal Care and Use (CACU) of the Lee Gil Ya Cancer and Diabetes Institute of Gachon University. All animal protocols described in this study were approved by the CACU animal center ethics board. Eight-week-old male HRM-2 mice (20–25 g) were randomly separated into four mice per cage and housed under a temperature-controlled environment with a 12-h light/dark cycle and free access to food and water. The mice were regularly exposed to UV-B for 5 min every 2 d for 10 d and then for 5 min every day for the following 3 d [43]. Subsequently, the mice were RF irradiated (2 MHz, 10 W, 100 ms) and then exposed to UV-B every 2 d for 28 d. Skin tissues were collected after 6 h, 12 h, 24 h, and 28 d of RF irradiation (Figure 1A).

### 4.4. Sample Preparation

#### 4.4.1. RNA Extraction and cDNA Synthesis

Total RNA was isolated using RNAiso Plus (Cat. 910; Takara, Shiga, Japan) according to the manufacturer’s instructions. The extracted RNA pellets were washed with a high-quality 70% solution. The pellets were dried and dissolved in 30 µL of diethyl pyrocarbonate-treated water. The extracted RNA was quantified using a Nanodrop 2000 (Thermo Fisher Scientific, Waltham, MA, USA) and was synthesized using a cDNA synthesis kit (Cat. 6110A; Takara) according to the manufacturer’s instructions.

#### 4.4.2. Paraffin-Embedded Tissue Sectioning

The skin tissues were stored in 4% paraformaldehyde (Cat. 16005; Sigma Aldrich, St. Louis, MO, USA) at 4 °C for 12 h. The fixed tissues were washed for 12 h for embedding and were processed into paraffin blocks using a tissue processor (Thermo Fisher Scientific). The blocks were cut into 7 µm-thick sections using a microtome (Leica, Wetzlar, Germany) and dried at 45 °C overnight. The paraffin blocks were passed through xylene and four concentrations of ethanol (100%, 95%, 80%, and 70%) to prepare them for staining.

### 4.5. Quantitative Real-Time Polymerase Chain Reaction

The synthesized cDNA was used for quantitative real time polymerase chain reaction (qRT–PCR). The qRT–PCR mixtures contained 5 µL of SYBR Green reagent (Cat. #RR430A, Takara), 1 µg of cDNA template, and 10 pmol of primer and were dispensed in each well of a 384 well multiplate and then analyzed using the CFX386 Touch Real-Time PCR Detection System (Bio–Rad, Hercules, CA, USA). The validated genes are listed in Table S1.

### 4.6. Tyrosinase Activity Analysis

Tyrosinase activity was determined in each group using the appropriate kit (Cat. ab252899; Abcam, Cambridge, UK) in accordance with the manufacturer’s instructions.

### 4.7. Immunohistochemistry Using 3,3-Diaminobenzidine

To block endogenous peroxidase, skin tissue slides were incubated in 0.3% hydrogen peroxide for 30 min at room temperature. The tissues were rinsed three times with phosphate-buffered saline (PBS) and then incubated with primary antibodies in normal serum for 24 h at 4 °C. Validated antibodies are listed in Table S2. The slides were washed three times with PBS and incubated in biotinylated secondary antibody using the ABC kit (Cat. 6100; Vector Laboratories Inc., Burlingame, CA, USA) for 2 h at room temperature and then were washed three times with PBS. The tissue slides were incubated with 3,3′-diaminobenzidine (Cat. D8001; Sigma Aldrich) for 15 min at room temperature and washed with running water. To stain nuclei, tissue slides were immersed in hematoxylin solution for 1 min at room temperature and then mounted with dibutylphthalate polystyrene xylene mounting solution (DPX, Cat. 06522; Sigma Aldrich). The stained tissues were photographed under an optical microscope (Olympus Optical Co., Tokyo, Japan) and analyzed with ImageJ software (NIH, Bethesda, MD, USA).

### 4.8. Fontana Masson Staining

The skin tissues were incubated in Fontana ammoniacal silver solution overnight at room temperature. The tissues were rinsed three times with distilled water and then incubated in hypo-osmotic solution for 3 min at room temperature. Afterward, the tissues were washed with distilled water and counterstained with neutral red stain for 5 min at room temperature. The tissues were then washed again with distilled water, dehydrated in absolute alcohol, and mounted for further observation.

### 4.9. Statistical Analysis

We performed nonparametric tests in this study, and the Kruskal–Wallis test was used to determine the statistical significance between three or four groups. When statistical significance was confirmed, the Mann–Whitney U test was used as a post hoc test for multiple comparisons by SPSS version 22 (IBM Corporation; Armonk, NY, USA). All results are represented as the mean ± standard deviation.

## Data Availability

All data are contained within the article.

## Supplementary Materials

The following are available online, Table S1: List of primers for qRT–PCR used in this study, Table S2: List of antibodies for immunohistochemistry used in this study.

## Sample Availability

Samples of the compounds are not available from the authors.
