# Supplementary material for: Radiofrequency Irradiation Mitigated UV-B-Induced Skin Pigmentation by Increasing Lymphangiogenesis
Source: Molecules. 2022 Jan 11;27(2):454. doi: 10.3390/molecules27020454 (PMC8780734; doi:10.3390/molecules27020454)
Supplement: Supplementary file 1 [file molecules-27-00454-s001.zip › molecules-1551523-supplementary.pdf]

Article

Radiofrequency Irradiation Mitigated UV-B-Induced Skin Pigmentation by Increasing Lymphangiogenesis

Hyoung Moon Kim [1,2,†], Seyeon Oh [2,†], Kyung-A Byun [1,2], Jin Young Yang [2], Hye Jin Sun [3], Miran Jang [3], Donghwan Kang [3], Kuk Hui Son [4,*] and Kyunghee Byun [1,2,*]

1 Department of Anatomy & Cell Biology, College of Medicine, Gachon University ,Incheon 21936, Republic of Korea; drmac12@me.com (H.M.K.); kabyun95@gmail.com (K.-A.B.)

2 Functional Cellular Networks Laboratory, College of Medicine, Department of Medicine, Graduate School and Lee Gil Ya Cancer and Diabetes Institute, Gachon University, Incheon 21999, Republic of Korea; seyeon8965@gmail.com (S.O.); roswellgirl111@gmail.com (J.Y.Y.)

3 Jeisys Medical Inc., Seoul, 08501, Republic of Korea; sunhj@jeisys.com (H.J.S.); lyla@jeisys.com (M.J.); kang@jeisys.com (D.K.)

4 Department of Thoracic and Cardiovascular Surgery, Gil Medical Center, Gachon University, Incheon 21565, Republic of Korea

† These authors contributed equally to this study

* Correspondence: dr632@gilhospital.com; Tel.: +82-32-460-3666 (K.H.S.); khbyun1@gachon.ac.kr; Tel.: +82-32-899-6511 (K.B.)

Supplementary Tables

Table S1. List of primers for qRT-PCR used in this study.

Gene	Species	Primers	
Hsp90	mouse	Forward	5′-CTA CCA GAG GAC GAG GAA GAG A-3′
		Reverse	5′-AAG ATC TCC TTC ATG AGC TTG C-3′
Braf		Forward	5′-AAG TCG GAT GAA AAC ACT TGG T-3′
		Reverse	5′-CTG TCC CAC TGT AAT CTG TCC A-3′
Mek		Forward	5′-ACC GAG GCT TCT TCC TTC TC-3′
		Reverse	5′-GCC CTT CTT CAC CTT CTC TTT T-3′
Erk		Forward	5′-TTC AAG TTT GAC ATG GAG TTG G-3′
		Reverse	5′-GAT CTG TAT CCT GGC TGG AAT C-3′
Actb		Forward	5'-CCG TAA AGA CCT CTA TGC CAA C-3′
		Reverse	5'-GCA GTA ATC TCC TTC TGC ATC C-3′
HSP90	human	Forward	5′-GTT GAA AAG GTG GTT GTG TCA A-3′
		Reverse	5′-TCA TGA TTC TCT CCA TGT TTG C-3′
BRAF		Forward	5′-CAG CTC CCA ATG TGC ATA TAA A-3′
		Reverse	5′-ACC ACG AAA TCC TTG GTC TCT A-3′
MEK		Forward	5′-AGG TCC TCA CCA AAG TCC TAC A-3′
		Reverse	5′-AGT CTT TGA CGA AGG ACT GGA A-3′
ERK		Forward	5′-GGA ATT GGA TGA CTT GCC TAA G-3′
		Reverse	5′-GAT CTG TAT CCT GGC TGG AAT C-3′
LYVE-1		Forward	5′-GCT AGT GCT TGC TCT CCT CTT C-3′
		Reverse	5′-AAG GCC TTC ACA TAC CTT TTG A-3′
VE-cadherin		Forward	5′-GAT CTC CGC AAT AGA CAA GGA C-3′
		Reverse	5′-TCC GTG AGG GTA AAG TTG TTC T-3′
ACTB		Forward	5′-GGG ACC TGA CTG ACT ACC TCA T-3′
		Reverse	5′-CCT TAA TGT CAC GCA CGA TTT-3′

Table S2. List of antibodies for immunohistochemistry used in this study.

Antigen (host)	Company	Catalog no.	Dilution rate
VEGF-C (Rabbit)	Invitrogen	PA5-29772	1:100
VEGFR 3 (Mouse)	Santa cruz biotechnology	sc-514825	1:50
PI3K (Mouse)	Santa cruz biotechnology	sc-376112	1:100
pAKT 1/2 (Mouse)	Santa cruz biotechnology	sc-514032	1:50
pERK 1/2 (Rabbit)	Invitrogen	PA5-37824	1:200
LYVE-1 (Rabbit)	LSBio	LS-B10511	1:100
VE-cadherin (Goat)	Santa cruz biotechnology	sc-6458	1:100
CD68 (Mouse)	Santa cruz biotechnology	sc-70761	1:100
